# Treatment of malignant esophageal stricture or esophageal leakage with a Niti-S™ covered anti-reflux stent is safe and efficient

**DOI:** 10.1007/s00423-026-04111-5

**Published:** 2026-06-23

**Authors:** Lisa Gensthaler, Johanna Bauer, Dagmar Kollmann, Milena Bologheanu, Joy Feka, Ivan Kristo, Gerd Jomrich, Erwin Rieder, Reza Asari, Sebastian F. Schoppmann

**Affiliations:** 1 Department of General Surgery, Division of Visceral Surgery, Comprehensive Cancer Center Vienna, Upper GI- Service, Medical University of Vienna, Spitalgasse 23, Vienna, 1090 Austria; 2 Department of Thoracic Surgery, Medical University of Vienna, Spitalgasse 23, Vienna, 1090 Austria

**Keywords:** Esophageal cancer, SEMS, Endoscopic stent placement, Anti-reflux stent

## Abstract

**Background:**

Self-expanding metal stents (SEMS) are a palliative treatment option for malignant esophageal strictures causing dysphagia. Retrosternal pain, reflux-related symptoms and migration remain relevant limitations. SEMS with an anti-reflux valve (SEMS-ART; Niti-S**™** esophageal covered stent, anti-reflux type, TaeWoong Medical) may reduce these limitations but clinical data remain limited. This study evaluates safety, tolerability and clinical performance of SEMS-ART in malignant esophageal stricture and leakage.

**Materials and methods:**

All patients treated with SEMS-ART for malignant esophageal stricture or leakage at the Medical University of Vienna, Department of General Surgery, between 01/2016 and 04/2021 were included into this retrospective analysis. Parameters regarding patients’ safety, compliance and associated comorbidities like reflux and dysphagia were analyzed.

**Results:**

Twenty patients were included. The cohort was predominantly male (90.0%) with a mean age of 65.3 years. Advanced esophageal cancer UICC stage III–IV was present in 75.0%. Technical success was achieved in all patients (100%). Adequate stent expansion was confirmed in all patients, while correct positioning and normal contrast passage were observed in 95%. Reflux, Dysphagia and Regurgitation resolved in all cases after SEMS insertion (*p* < 0.001). Dislocation occurred in 15.0% (*n* = 3) and primarily managed endoscopically. Mean SEMS dwell time was 88.3 days. 30-day SEMS-related safety was 95.0%, and no SEMS-related mortality occurred within the observational period.

**Conclusion:**

This small case series demonstrates high technical success of SEMS-ART, supporting feasibility and an acceptable safety profile on migration and symptom relief. These findings support the use of SEMS-ART in selected patients with malignant esophageal stricture and should be considered hypothesis-generating, supporting further prospective comparative studies.

## Background

**Fig. 1:**
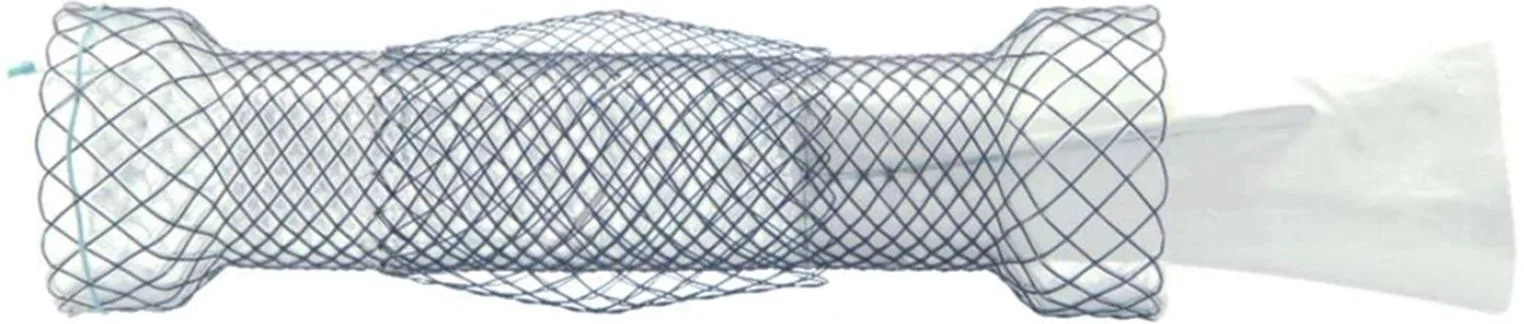
Niti-STM esophageal covered stent [Double Anti Reflux-Type], TaeWoong Medical

Esophageal Cancer (EC) represents a major concern for global health, ranking currently as the sixth leading cause of cancer-related mortality worldwide [1, 2]. In 2020, more than 604,100 new cases and 544,000 deaths were reported globally [3]. Particularly in high-income regions such as North America and Europe, incidence of adenocarcinoma of the esophagogastric junction (AEG) has increased steadily, closely associated with lifestyle-related risk factors such as gastroesophageal reflux disease (GERD) and obesity [4]. Because it is frequently diagnosed at an advanced stage, it remains associated with high morbidity and poor long-term survival [5]. Dysphagia occurs in 70–90% and is the most common and distressing symptom, leading to malnutrition, weight loss, impaired quality of life and reduced tolerance to systemic therapy. Therefore, early detection and specialized treatment in high-volume centers are essential for improving patient’s outcome [6]. In patients with malignant esophageal stricture and locally advanced- or metastatic disease, palliative restoration of luminal patency is an essential symptomatic treatment [7]. Therefore, endoscopic placement of self-expandable metal stents (SEMS) is well-established and provides sufficient and rapid symptom relief in 80–95% of patients [8–11] and closure of leaks and fistulas also [12]. SEMS placement enables the majority of patients to resume oral intake and consecutively significantly improving quality of life in patients with advances disease and limited life expectancy. Fully covered SEMS are preferred in these patients with expectedly long dwell time to prevent tumor ingrowth and facilitate removal if necessary [13–15]. Nevertheless, stent-related adverse events remain clinically relevant and include retrosternal pain (20–50%) and discomfort, GERD, bleeding (2–10%), perforation (< 5%), tumor overgrowth and stent migration (10–30%), particularly when the device is placed at the esophagogastric junction (EGJ) [8, 9, 16]. Especially, reflux-related symptoms such as GERD, regurgitation and aspiration might not only comprise patients’ quality of life substantially. They are reported in 30–60% of patients with conventional covered SEMS but also might lead to recurrent aspiration or pneumonia [17]. Therefore, dedicated SEMS, such as the CE-certified Niti-S™ Esophageal Covered Stent Anti-Reflux Type (ART) (Taewoong Medical, Seoul, Korea), incorporating an anti-reflux valve (ARV) mechanism have been developed to maintain luminal patency for oral food intake whilst restoring the barrier function and reducing reflux-related complications in locally advanced EC (Fig. 1) [18].

This retrospective single-center case series, conducted at the Department for General Surgery, Medical University of Vienna (MUV), includes data of 20 consecutive patients with dysphagia and malignant esophageal stricture, treated with SEMS-ART. It aims to assess the tolerability and safety of SEMS-ART with particular emphasis on dislocation and migration, GERD and SEMS associated complications during the observational period. Although several randomized controlled trials have reported comparable outcomes between conventional covered SEMS and anti-reflux SEMS, real-world data on safety, tolerability and clinical performance of currently available anti-reflux stent systems remain limited [19]. Therefore, observational data from specialized centers may provide complementary information regarding feasibility, symptom control and device-related complications in daily clinical practice. Findings of this analysis shall support identifying potential risks and guide future prospective studies. It aims to define the role of SEMS-ARV in optimizing individualized palliative care for patients with malignant esophageal strictures.

## Material & methods

All patients with malignant esophageal stricture and dysphagia, who had undergone endoscopic treatment with SEMS-ART using the Niti-S™ Esophageal Covered Stent Anti-Reflux Type (Taewoong Medical, Seoul, Korea), at the Department for General Surgery, MUV, between 01/2016 and 04/2021 were included in this retrospective case series. Data used for this study are routinely assessed during patients’ medical treatment. Patient’s data were collected retrospectively, based on prospectively maintained databases and pseudonymized regarding standards of good scientific practice. This analysis was approved by the local ethics review committee (Ethics committee, MUV, Nr. 1277/2022). All endoscopic interventions were performed by the same team, specialized in Upper GI Surgery, performing surgery as well as endoscopy and SEMS placement themselves.

### Patient characteristics

Basic patient characteristics such as sex, age, BMI, and comorbidities and preoperative symptoms were collected and evaluated. Date of diagnosis and further therapeutic procedure (systemic therapy, irradiation, surgery, endoscopy) was evaluated as well. All patients included in this analysis received SEMS-ART due to dysphagia and locally advanced esophageal malignancy, stricture after local irradiation of EC or metastatic spread such as esophagotracheal- or esophagoaortic fistula. In selected cases, patients with EC received SEMS-ART postoperatively in case of leakage. Tumor entity and Staging regarding the UICC Classification were evaluated as well. Index Surgery was endoscopic stent placement at the MUV. Persistent symptoms and necessity for reinterventions were evaluated during patients’ follow-up visits. Mortality was defined as patients’ death within 90 days since the index SEMS placement and afterwards.

### Clinical characteristics

Perioperative data such as onset of symptoms, defined as the period between stent placement and the development of symptoms suggesting dislocation or migration were evaluated. Symptom assessment was performed retrospectively based on routinely documented clinical evaluations during inpatient treatment and outpatient follow-up visits. No validated dysphagia score, reflux or quality-of-life questionnaire was prospectively documented during the study period in all consecutive patients. Efficacy of SEMS placement was evaluated at the first postoperative day via contrast swallow x-ray and clinically afterwards. Technical success was defined as successful deployment of the SEMS at the intended target location with adequate expansion confirmed radiologically after implantation. Correct positioning and normal passage of contrast agent was evaluated as well.

Furthermore, during postinterventional observation, contrast swallow x-ray was performed where applicable before SEMS replacement or removal or if any novel symptoms occurred.

### Statistical Evaluation

Patient population was characterized by conducting descriptive statistics for pre- and postoperative parameters. Categorical variables were presented as numbers (n) and proportions (%), continuous variables as median with range or mean with standard deviation. Categorical variables were compared using the Fischer exact or the Pearson x^2^ tests, where appropriate. Clinical pre- and postoperative parameters were correlated, using one-way Anova and students’ t-test and chi-square tests, if appropriate. P-values < 0.05 were considered statistically significant. SPSS version 25 for Mac (IBM, Armonk, New York, USA) was used for statistical calculations.

## Results

### Patient characteristics

Within an observational period of 5 years (01/2016-04/2021), 20 patients presenting with dysphagia due to malignant esophageal stricture or associated complications were treated symptomatically by endoscopic placement of SEMS-ART (Niti-S™ Esophageal Covered Stent Anti-Reflux Type) at the Department for General Surgery, MUV. The cohort was predominantly male (*n* = 18/20; 90.0%) with a mean age of 65.3 (Min-Max:45–85) years. Median BMI at the time of first SEMS placement was 21.6 kg/m^2^ (IQR: 20.6–25.4 kg/m^2^). Patients presented with relevant comorbidities such cardiovascular disease (CVD; *n* = 11/20; 55.0%), chronic obstructive pulmonary disease (COPD; *n* = 2/20;10.0%), liver cirrhosis (*n* = 4/20; 20.0%), malignancy at another site (*n* = 2/20; 10.0%), nicotine abuse (*n* = 12/20; 60.0%) and pathologic alcohol consumption (*n* = 4/20; 20%). AEG was the primary tumor entity in 15 cases (75.0%), while in 5 patients (25.0%) squamous cell cancer (SCC) had been detected. In 15 cases (75.0%), EC was diagnosed at advanced UICC Stage 3 or 4 with positive lymph node involvement in 15 cases (75.0%) and metastatic spread in 9 cases (45.0%). All but one patient (*n* = 19) experienced an esophageal stenosis due to advanced tumor disease, one patient (5.0%) with a postoperative leak after Ivor-Lewis abdominothoracic esophagectomy (IL) was included also. Preoperative dysphagia and concomitant weight loss was frequent and identified in 17 (85.0%) and 19 cases (95.0%). GERD-like symptoms and regurgitation were described in 16 (80.0%) and 17 (85.0%) patients. Further details regarding patient characteristics are provided in Table 1.

**Table 1:** Patients’ and clinical characteristics

	n (%, Min-Max or IQR)
Sex	
Male	18 (90.0%)
Female	2 (20.0%)
Mean age (years, min-max)	65.3 (45–85)
Median BMI (kg/m^2^)	21.6 kg/m^2^ (20.6–25.4)
Comorbidities at the time of diagnosis	
Cardiovascular Disease (art HTN, KHK, infarction,…)	11 (55.0%)
COPD	2 (10.0%)
Nicotine abuse	12 (60.0%)
Pathologic alcohol consumption	4 (20.0%)
Cirrhosis	4 (20.0%)
Malignancy elsewhere	2 (10.0%)
Tumorentity	
Squamous Cell Cancer (ESCC)	5 (25.0%)
Adenocarcinoma (AEG)	15 (75.0%)
pT	
1b	1 (5.0%)
2	3 (15.0%)
3	6 (30.0%)
4	9 (45.0%)
pN	
+	15 (75.0%)
-	5 (25.0%)
pM	
+	9 (30.0%)
Liver	2 (10.0%)
Lung	3 (15.0%)
Peritoneum	1 (5.0%)
Infiltration thoracic Aorta	2 (10.0%)
Infiltration Trachea	2 (10.0%)
Grading	
2	6 (30.0%)
3	12 (60.0%)
UICC Staging	
1	3 (15.0%)
2	2 (10.0%)
3	4 (20.0%)
4	11 (55.0%)
Preoperative Symptoms	
Weight loss	19 (95.0%)
Dysphagia	17 (85.0%)
Reflux	16 (80.0%)
Regurgitation	17 (85.0%)

IQR Interquartile-Range, *BMI *(Body Mass Index), *ESCC* Esophageal Squamous Cell Cancer, *AEG* Adenocarcinoma of the Esophagogastric Junction, *Range days* Median (Minimum-Maximum)

### SEMS placement and clinical course

Therapeutic strategy was primarily a combination of neoadjuvant/palliative Chemo(-radio) therapy and SEMS placement (*n* = 14; 70%). Standalone SEMS and SEMS after Radiation was indicated in 2 cases (10%) each. 3 patients did receive Ivor Lewis (IL) esophagectomy and 2 patients a laparoscopic exploration. Primary Indication for SEMS was Dysphagia in 15 cases (75.0%), perforation and postsurgical leakage in 2 cases (10.0%) and Fistula in 3 cases (15.0%). Correct SEMS deployment was observed in all patients by contrast swallow x-ray on postoperative day 1 after insertion, demonstrating adequate stent expansion (*n* = 20; 100.0%), positioning (*n* = 19/20; 95.0%) and normal contrast passage (*n* = 19/20; 95%). One patient demonstrated delayed contrast passage despite adequate stent expansion and positioning; however, the patient remained clinically stable and did not require further intervention. During postinterventional follow-up, retrosternal pain was documented in 3 cases (15.0%), while reflux (*p* < 0.001), dysphagia (*p* < 0.001) and regurgitation (*p* < 0.001) resolved in all patients. SEMS dislocation occurred in 3 cases (15.0%) during follow-up, requiring endoscopic repositioning in 2 case (10.0%) and surgical intervention with laparotomy and enterotomy in 1 case (5.0%). Detailed information regarding therapeutic procedure and outcome is shown in Table 2.

**Table 2:** Therapeutic procedure & outcome

	n (%, IQR; min-max; p)
Therapeutic strategy	
Standalone SEMS	2 (10.0%)
Combination of neoadjuvant Chemo(-Radiatio)+SEMS	14 (70.0%)
FLOT/FOLFOX/FOLFIRI	8 (40.0%)
CROSS	4 (20.0%)
Other	2 (10.0%)
Radiation alone	2 (10.0%)
Surgical Intervention	5 (25.0%)
IL	3 (15.0%)
Laparoscopic exploration	2 (10.0%)
Indication for SEMS	
Dysphagia	15 (75.0%)
- Malignant stricture	11 (55.0%)
- Treatment effect (post-irradiation or surgery)	4 (20.0%)
Leakage	2 (10.0%)
- after surgical Intervention (IL)	1 (5.0%)
- after Perforation	1 (5.0%)
Fistula (Esophagobronchial/Esophagoaortal)	3 (15.0%)
SEMS on contrast swallow X-ray at POD 1	20 (100%)
Adequate expansion	20 (100%)
Normal contrast passage	19 (95.0%)
Adequate Positioning	19 (95.0%)
Clinical course after SEMS placement	
Retrosternal Pain	3 (15.0%)
Dysphagia	0 (0.0%; *p* < 0.001)
Reflux	0 (0.0%; *p* < 0.001)
Regurgitation	0 (0.0%; *p* < 0.001)
SEMS Dislocation	3 (15.0%)
Endoscopic repositioning	2 (10.0%)
Laparotomy	1 (5.0%)
Mean SEMS dwell time (days)	88.25 (IQR:18.8–117.0)
Number of SEMS per Patient (mean; min-max)	1.65 (1–10)
SEMS safety < 30 days after SEMS placement	19 (95.0%)
Follow-up (median)	205 (IQR:25–425)
Death during Follow-up	15 (75.0%)
Mortality	
<90 days after SEMS placement	4 (20.0%)
>90 days after SEMS placement	11 (55.0%)

*IL* Ivor Lewis Esophagectomy, *POD* Postoperative Day 1, *SEMS* Self expanding metal stent, *ARV* Anti-reflux Valve, *Range days* Median (Minimum-Maximum)

### Follow-up and outcome

**Fig. 2:**
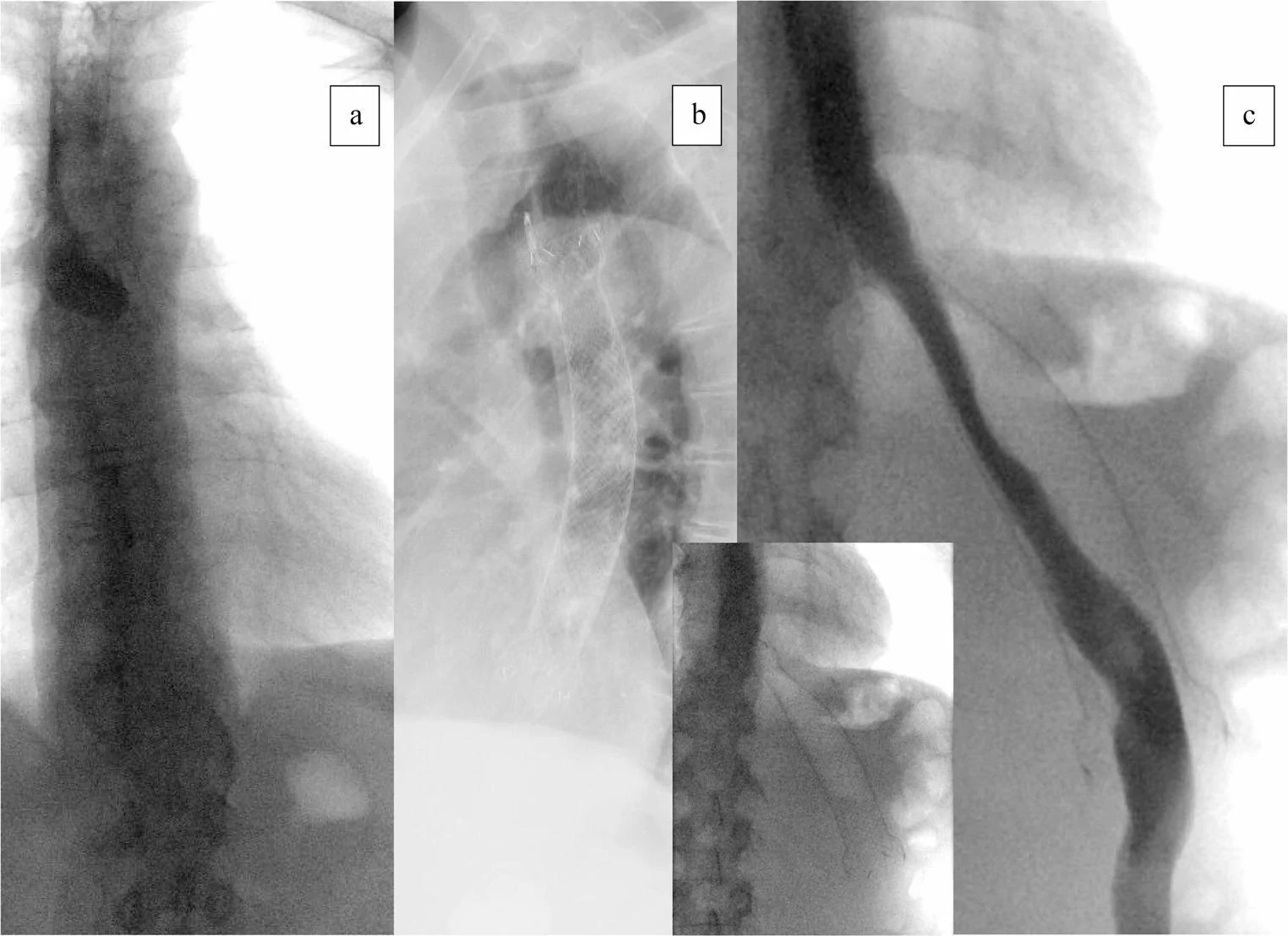
Contrast agent clearance before (**a**) and after (**b**,**c**) SEMS-ART insertion via x-ray

**Fig. 3:**
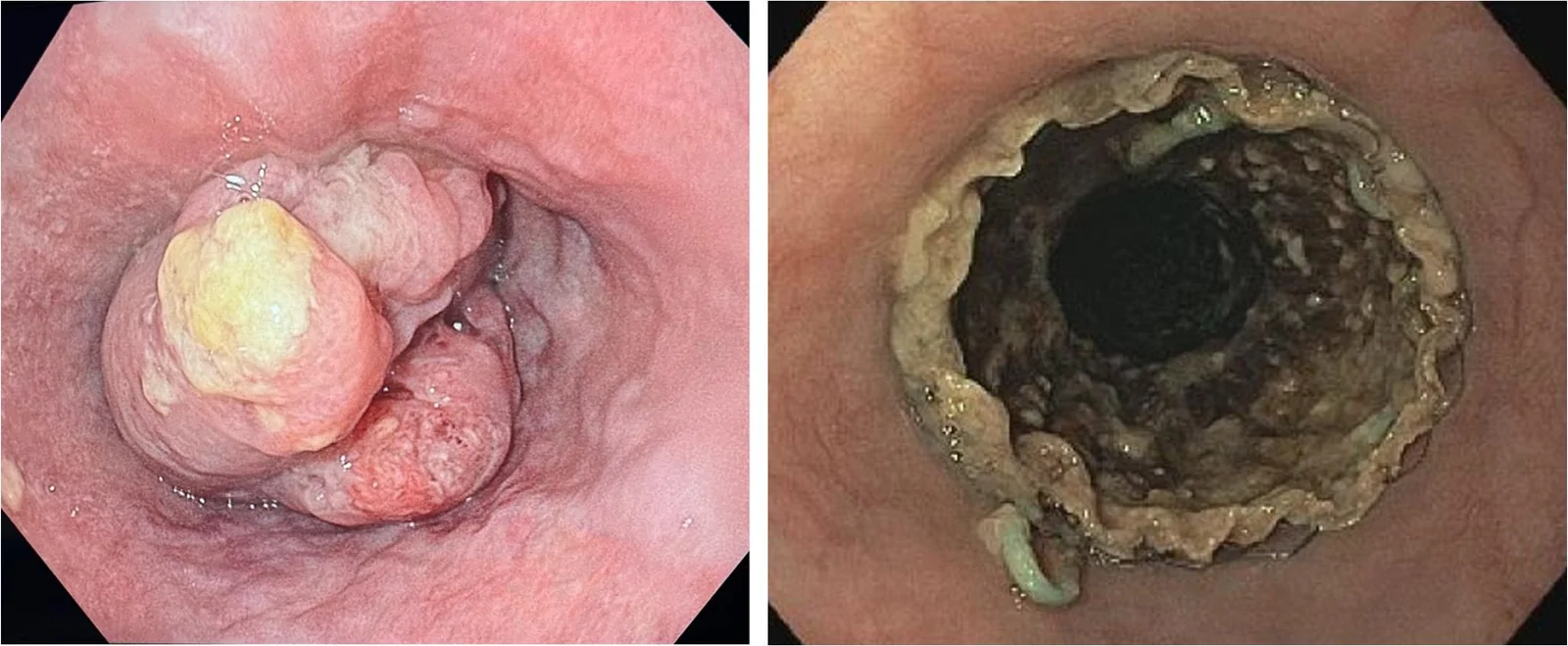
Endoscopic results before and after SEMS-ART insertion

Median follow up was 205 days (IQR:24–425 days). During the entire observational period, 15 patients died due to advanced disease and the palliative setting. In all cases, no SEMS related mortality was observed. Mean SEMS dwell time was 88.3 days (IQR:18.8–117.0), with a mean number of 1.65 (1–10) SEMS per patient. SEMS-related safety within the first 30 days after implantation was documented in 19 patients (95.0%). More detailed information is shown in Table 2. Figures 2 and 3 illustrate an exemplary case of locally advanced EC before and after successful SEMS-ARV therapy.

## Discussion

### Patient characteristics

This retrospective case series reports outcomes of SEMS-ART placement in a cohort of patients with malignant esophageal strictures, treated predominantly in a palliative setting. The majority of patients presented with advanced tumor stages at the time of endoscopic intervention, reflected by the high proportion of UICC stage III-IV disease, frequent lymph node involvement, metastatic spread and esophageal fistula, involving aorta and trachea. This cohort reflects a typical palliative population with advanced esophageal malignancy [1, 3, 5]. These findings are consistent with established indications for SEMS therapy and comparable to previously published observational cohort studies, where advanced disease predominates due to the palliative indication of stent therapy [7, 20]. Primary symptoms were dysphagia, weight loss, regurgitation and GERD-like symptoms which are substantial associated with low median BMI, underlining the impaired nutritional status of these patients. Similar baseline rates have been reported in large observational SEMS series, illustrating the advanced nutritional impairment, typical of patients referred for palliative stent therapy [13]. Therefore, SEMS primarily aims to achieve rapid symptom relief and sufficient oral food intake, rather than oncologic disease control. Data on clinical effect on dysphagia relief are promising, but data from meta-analyses and randomized studies are divergent, showing inconsistent effect on reflux control with no clear superiority compared to conventional SEMS [17]. Previously published small case series on SEMS-ART on the other hand have reported favorable technical success, effective dysphagia relief and low GERD and migration rates, data on tolerability and safety in daily clinical practice remain scarce [8]. Therefore, the aim of the present study was to provide real-world data regarding feasibility, safety and symptom control of SEMS-ART in a consecutive cohort of patients treated in a tertiary referral center, not to demonstrate superiority over conventional SEMS [19].

### SEMS placement and clinical course

SEMS-ART placement, using the Niti-S™ Esophageal Covered Stent Anti-Reflux Type (Taewoong Medical, Seoul, Korea), was technically successful in all 20 patients (100%), although EC was obstructive and locally advanced and therefore adequate placement challenging, in most cases. This is consistent with the high technical success rates (90–100%) reported across multiple SEMS studies [13, 14]. Precise and proper placement was carried out under radiologic guidance and verified endoscopically after deployment. Radiologic evaluation, routinely performed on postoperative day one, confirmed adequate stent expansion in 100% of cases, with correct positioning and normal contrast passage in 95%. These findings underline reliable results regarding efficacy and intuitive management of the SEMS-ART in routine clinical practice [18]. Dysphagia resolved in all patients following SEMS placement, confirming the high clinical efficacy of the stent in restoring luminal patency and symptom control in malignant EC. These findings are consistent with previously published randomized trials demonstrating that SEMS placement provides rapid and effective dysphagia relief in the majority of patients with malignant esophageal obstruction [15, 21, 22]. Interestingly, comparative studies have shown no difference on dysphagia relief between conventional SEMS and SEMS with an integrated ARV [15, 21, 22]. Quality of life outcomes following SEMS are generally favorable, with most patients reporting improved oral intake and reduced symptom burden after intervention and also have shown comparable improvements between standard SEMS and SEMS-ART, suggesting that both designs provide effective palliation in advanced EC [8, 15, 22]. Similarly, reflux symptoms and regurgitation, which were highly prevalent before intervention, were no longer observed during postinterventional follow-up. This observation is of special interest in patients with distal EC or EC at the EGJ. Since published data typically report significant improvement in 80–95% of patients, results of this analysis support the intended effect of the integrated ARV [14]. Conventional fully covered SEMS are associated with reflux rates of up to 30–60% in distal esophageal tumors [11]. In contrast, van Overbeke et al. reported a low incidence of reflux symptoms using the Niti-S™ double anti-reflux design [18]. The absence of clinically relevant reflux in our cohort aligns with these findings and supports the intended functional benefit of the anti-reflux valve [14, 18]. From a technical perspective, both malignant dysphagia and esophageal leakage may be sufficiently treated with various types of covered esophageal stents [12, 23]. However, in palliative patients with an expected prolonged SEMS dwell time and an intended limited number of stent exchanges, reflux-related symptoms may significantly compromise daily quality of life. This finding is consistent with the established role of covered SEMS as first-line palliative treatment and aligns with data from the post-market registry reported by van Overbeke et al., who demonstrated significant dysphagia relief following implantation of Niti-S™ Esophageal Covered Stent (Anti-Reflux Type, TaeWoong Medical) [18], . In this specific subgroup, the use of a SEMS-ART may therefore offer a clinically relevant advantage by reducing reflux-associated symptoms, particularly in those with distal EC or AEG tumors [8, 15, 18]. Stent dislocation occurred in a limited number of cases (*n* = 3;15.0%) and could be managed endoscopically in most patients. The observed migration rate is within the range reported for fully covered SEMS and comparable to migration rates described in post-market surveillance data for SEMS-ART [24]. No technical failures requiring emergency intervention was observed.

### Safety and tolerability

Safety within the first 30 days after SEMS placement was high (95%), with no observed SEMS-related mortality and no severe adverse events such as perforation and major bleeding. Published complication rates for conventional SEMS include migration in 23% to 48%, chest pain in 20–50%, and severe adverse events below 10% [24–26]. Since comparative studies on SEMS with and without ARV are similar, the risk for migration is mainly influenced by SEMS covering characteristics and tumor morphology. Other mentioned complications are similar between differing SEMS models, supporting SEMS as preferred treatment modality of malignant esophageal obstruction, while acknowledging ongoing innovation in SEMS design to optimize patient outcomes while reducing complications [25, 26].

In our cohort, stent dislocation occurred in 15%, which falls within the expected range for fully covered SEMS [13, 14]. These finding support the procedural safety of SEMS-ART in a fragile oncologic cohort and is in accordance with previously published post-market clinical follow-up studies. As reported earlier, retrosternal pain and discomfort are the most common but usually manageable side effects of SEMS therapy. Importantly, GERD related symptoms resolved completely and were not observed after SEMS placement in this cohort. This supports the intended function of the integrated ARV and aligns with results by van Overbeke et al. who reported low incidence of GERD after SEMS-ART [18]. The main drawback of the present study is the lack of any control group with conventional covered SEMS. However, the absence of clinically relevant GERD symptoms suggests a potential benefit to the ARV in distal EC or AEG. Tolerability was generally good, whilst retrosternal pain occurred in few patients and represented the main reason (*n* = 3; 15.0%) for unplanned SEMS removal. During SEMS removal, no major associated complications were observed.

### Follow-up and outcome

Median follow-up duration was 205 days (IQR:25–425) and allowed assessment of mid-term outcomes. Mortality during follow-up reached 75%, reflecting the advanced oncologic stage and palliative intent rather than SEMS-related complications. Most deaths occurred beyond 90 days after SEMS placement and were associated to tumor progression. Mean SEMS dwell time was 88 days, consistent with previously reported median indwelling times of 1–4 months in palliative cohorts [9, 13, 23]. The need for more than one SEMS in some patients reflects tumor progression rather than device failure. Nevertheless, sustained relief of dysphagia was achieved despite disease progression, supporting the role of SEMS as an effective palliative measure.

### Limitations

This study is limited due to its retrospective design, the single-center study setting and small sample size. The absence of a control group of patients treated with conventional covered SEMS without ARV is another limitation which must be pointed out. Consequently, symptom improvement cannot be attributed specifically to the anti-reflux valve design but may reflect the established palliative effect of SEMS placement in general. Furthermore, symptom assessment was based on routine clinical documentation in most patients, rather than a validated symptom score or quality-of-life questionnaire and therefore not available for comparative analysis. Consequently, the findings should be interpreted as hypothesis-generating.

## Conclusion

The findings of this small retrospective case series suggest that SEMS-ART (Niti-S™ Esophageal Covered Stent Anti-Reflux Type, TaeWoong Medical) placement is technically feasible and associated with an acceptable safety profile and favorable clinical outcomes in selected patients with malignant esophageal strictures and leakage. Improvement of dysphagia and GERD-related symptoms was observed in this fragile palliative patient cohort. Nevertheless, given the retrospective design, limited sample size and absence of a control group, these findings should be considered hypothesis-generating and warrant further evaluation in prospective comparative studies.

## Data Availability

The data supporting the findings of this study are available on request from the corresponding author S.F.S.

## Declarations

### Data access statement

The data supporting the findings of this study are available on request from the corresponding author S.F.S.
